# The haplotype-resolved T2T reference genome highlights structural variation underlying agronomic traits of melon

**DOI:** 10.1093/hr/uhad182

**Published:** 2023-08-28

**Authors:** Guoli Li, Lingli Tang, Yuhua He, Yongyang Xu, Abdelhafid Bendahmane, Jordi Garcia-Mas, Tao Lin, Guangwei Zhao

**Affiliations:** National Key Laboratory for Germplasm Innovation & Utilization of Horticultural Crops, Zhengzhou Fruit Research Institute, Chinese Academy of Agricultural Sciences, Zhengzhou, Henan 450009, China; China Agricultural University, College of Horticulture, Beijing 100193, China; National Key Laboratory for Germplasm Innovation & Utilization of Horticultural Crops, Zhengzhou Fruit Research Institute, Chinese Academy of Agricultural Sciences, Zhengzhou, Henan 450009, China; National Nanfan Research Institute (Sanya), Chinese Academy of Agricultural Sciences, Sanya, Hainan 572024, China; Zhongyuan Research Center, Chinese Academy of Agricultural Sciences, Xinxiang 453400, China; National Key Laboratory for Germplasm Innovation & Utilization of Horticultural Crops, Zhengzhou Fruit Research Institute, Chinese Academy of Agricultural Sciences, Zhengzhou, Henan 450009, China; National Nanfan Research Institute (Sanya), Chinese Academy of Agricultural Sciences, Sanya, Hainan 572024, China; National Key Laboratory for Germplasm Innovation & Utilization of Horticultural Crops, Zhengzhou Fruit Research Institute, Chinese Academy of Agricultural Sciences, Zhengzhou, Henan 450009, China; National Nanfan Research Institute (Sanya), Chinese Academy of Agricultural Sciences, Sanya, Hainan 572024, China; Institute of Plant Sciences Paris-Saclay (IPS2), INRAE, CNRS, University of Paris-Saclay, University of Evry, University of Paris-Diderot, Gif sur Yvette 91192, France; Centre for Research in Agricultural Genomics (CRAG) CSIC-IRTA-UAB-UB, Edifici CRAG, Campus UAB, Bellaterra, 08193 Barcelona, Spain; Institut de Recerca i Tecnologia Agroalimentàries (IRTA), Edifici CRAG, Campus UAB, Bellaterra, 08193 Barcelona, Spain; China Agricultural University, College of Horticulture, Beijing 100193, China; National Key Laboratory for Germplasm Innovation & Utilization of Horticultural Crops, Zhengzhou Fruit Research Institute, Chinese Academy of Agricultural Sciences, Zhengzhou, Henan 450009, China; National Nanfan Research Institute (Sanya), Chinese Academy of Agricultural Sciences, Sanya, Hainan 572024, China; Zhongyuan Research Center, Chinese Academy of Agricultural Sciences, Xinxiang 453400, China

## Abstract

Melon (*Cucumis melo* L.) is an important vegetable crop that has an extensive history of cultivation. However, the genome of wild and semi-wild melon types that can be used for the analysis of agronomic traits is not yet available. Here we report a chromosome-level T2T genome assembly for 821 (*C. melo ssp. agrestis* var. *acidulus*), a semi-wild melon with two haplotypes of ~373 Mb and ~364 Mb, respectively. Comparative genome analysis discovered a significant number of structural variants (SVs) between *melo* (*C. melo ssp. melo*) and *agrestis* (*C. melo ssp. agrestis*) genomes, including a copy number variation located in the ToLCNDV resistance locus on chromosome 11. Genome-wide association studies detected a significant signal associated with climacteric ripening and identified one candidate gene *CM_ac12g14720.1* (*CmABA2*), encoding a cytoplasmic short chain dehydrogenase/reductase, which controls the biosynthesis of abscisic acid. This study provides valuable genetic resources for future research on melon breeding.

## Introduction

Melon (*Cucumis melo* L., *2n* = 24) is an important crop cultivated worldwide, with more than 28 million tons produced in 2021 (Food and Agriculture Organization statistics, http://www.fao.org/). Melon has been classified into 16 varieties belonging to two subspecies, *C. melo ssp. agrestis* (hereafter *agrestis*) and *C. melo ssp. melo* (hereafter *melo*) [1]*.* A previous study suggested that melon has undergone three independent domestication events, one in Africa and two in India, and most of the modern melon accessions were domesticated from India [2]. Geographically, *agrestis* was cultivated in East Asia, whereas *melo* was cultivated worldwide, which resulted in diverse characteristics between the two subspecies. Independent domestication may have generated different genetic mechanisms for the same trait between the two subspecies, shaping genomic imprinting in their genomes.

Current genomic sequencing technologies offer powerful tools for crop breeding. To date, a few high-quality melon genomes have been reported, including two cultivated *agrestis* and three *melo* genomes [3–8]. The availability of newly developed assembly algorithms and software has enabled the assembly of telomere-to-telomere (T2T) genomes [9]. The first T2T genome of a *Cucurbitaceae* crop, watermelon, has been released, harboring eleven chromosomes without gaps [10]. Although several melon genomes with high quality and continuity have been assembled using different assembly strategies, a considerable fraction of the genome and a number of gaps still remain to be resolved. The two representative melon genomes CM4.0 [8] and Harukei-3 [6] contained 1169 and 94 gaps, respectively. Wild germplasm is an important genetic resource in crop breeding because of its high genetic diversity, which has been used extensively in rice, maize, and tomato [11–13]. In fact, cultivated melon accessions have been widely utilized, whereas genomes of wild and semi-wild melons have been used to a lesser extent.

Melon domestication has resulted in fruit quality and disease resistance differences between wild and cultivated melons [14]. In previous studies, many resistant accessions were evaluated and made it possible to identify potential disease-resistance loci in melon [15–17]. Additionally, because of presenting both climacteric and non-climacteric types, melon was considered as a unique model species to study fruit ripening [18]. In melon, several ethylene biosynthesis-related loci and genes have been identified, including *ETHQB3.5*, *ETHQV6.3*, and *ETHQV8.1*[19–25]. It is widely acknowledged that the ripening of climacteric fruits is dependent on ethylene, whereas in non-climacteric fruits as grape, strawberry, and cherry, abscisic acid (ABA) is considered as the major regulatory phytohormone during the ripening process [26–29].

A previous phylogenetic analysis demonstrated that *C. melo ssp. agrestis* var. *acidulus* belonged to the semi-wild group [2]. PI 313970 is an accession derived from *C. melo ssp. agrestis* var. *acidulus* native to India, which possess high resistance to *Cucurbit yellow stunting disorder virus* (CYSDV), *Cucurbit aphid-borne yellows virus* (CABYV), *Watermelon chlorotic stunt virus* (WmCSV), *Lettuce infectious yellows virus* (LIYV), *Cucurbit leaf crumple virus* (CuLCrV), and *Tomato Leaf curl new delhi virus* (ToLCNDV) [15, 17, 30, 31]. Additionally, the fruit of PI 313970 possesses excellent shelf life because of its non-climacteric fruit, which can be considered as an excellent accession to study fruit ripening [16, 32]. The 821 accession was derived from PI 313970 by self-fertilizing. In this study, we present a T2T genome assembly of 821, identifiying a large number of structural variations (SVs) between the *melo* and *agrestis* genomes and several resistance genes impacted by SVs by comparative analysis among seven available melon genomes. Based on the T2T-821 genome, we identified one signal significantly associated with climacteric ripening by genome-wide association study (GWAS). The study will provide an unprecedented opportunity for gene discovery and melon breeding.

## Results

### Genome sequencing, assembly, and annotation

**Figure 1:**
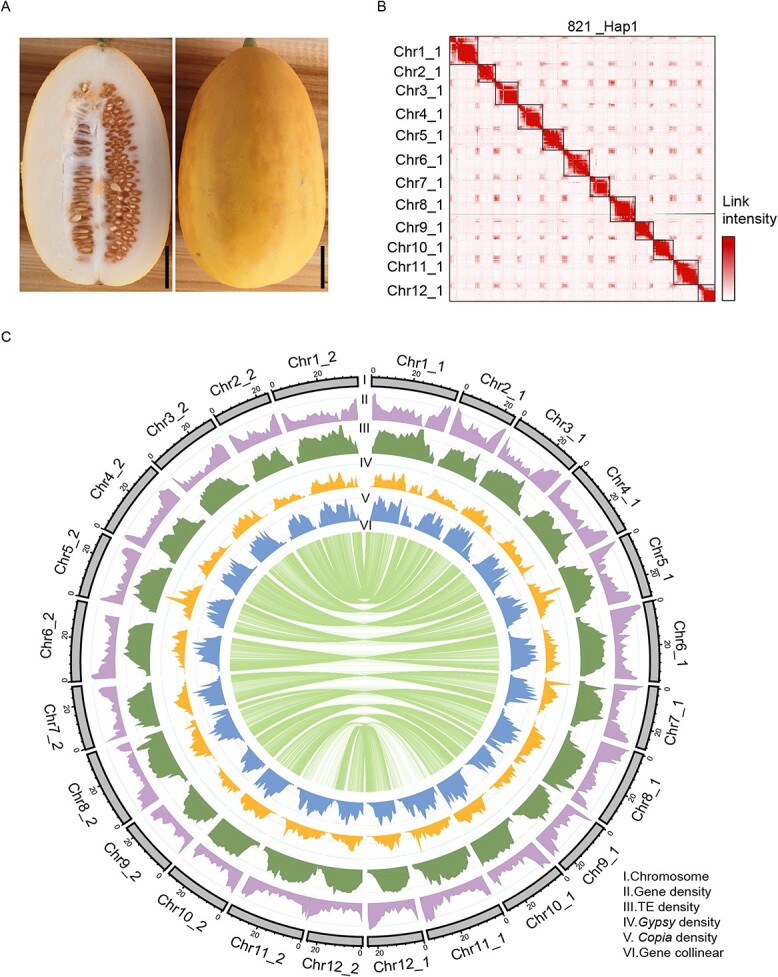
Phenotype, Hi-C map and Genomic landscape of 821. **A** The fruit of 821. Scale bar is 2.5 cm. **B** Hi-C map of the 12 chromosomes of the 821_Hap1 subgenome. The color in the heatmap represents the interaction intensity. **C** Genomic landscape of the 821 genome. I: Ideogram of the 12 chromosomes with scale in Mb. II: Gene density along each chromosome (number of genes per Mb). III: Repeat content along each chromosome (% nucleotides per Mb). IV: *Gypsy* retrotransposons content (% nucleotides per Mb). V: *Copia* retrotransposons content (% nucleotides per Mb). VI: Gene collinearity between the 821_Hap1 and 821_Hap2 subgenomes.

In this study, the representative semi-wild accession 821 was selected for genome assembly, which possesses high resistance to a variety of viral diseases (ToLCNDV, CYSDV, CABYV, WmCSV, and CuLCrV), high flesh firmness and long shelf life (Fig. 1A). A total of 31.83 Gb of PacBio HiFi reads (~79× genome coverage), 41.09 Gb of Hi-C clean reads (~115× genome coverage), and 33.77 Gb of Illumina reads (~84× genome coverage) were generated (Table S1, see online supplementary material). The k-mer analysis estimated that the size of the 821 genome was ~389 Mb with 0.358% heterozygosity (Fig.S1, see online supplementary material). Using the Hifiasm program [33], we assembled the contigs and produced an 882.10-Mb genome assembly containing 473 contigs (Table 1). Finally, the 821 genome was phased into two haplotypes with 24 chromosomes using the Hi-C data, including ~373.31 Mb (821_Hap1) with a contig N50 size of 10.26 Mb and ~364.05 Mb (821_Hap2) with a contig N50 size of 11.86 Mb (Fig. 1B and Table 1; Fig. S2, see online supplementary material). The assembly size was similar to that of previously published melon genomes (Table 1) [3–8].

**Table 1:** The assembly statistics of seven melon genomes

Assembly	821_Hap1	821_Hap2	CM4.0	IVF77	HS	CMiso1.1	Payzawat	Harukei-3
Size of 12 chromosomes (Mb)	373.31	364.05	357.73	330.77	359.41	361.86	363.79	370.16
Year	2022	2022	2019	2020	2020	2021	2019	2020
Number of chromosomes	12	12	12	12	12	12	12	12
Number of contigs	243	230	131	1698	298	236	882	112/1381
Contig N50 (Mb)	10.26	11.86	0.71	0.49	3.45	6.8	2.86	8.62
Contig L50	20	12	131		35		51	
Number of genes	27 685	27 258	28 299	27 073	28 897	33 936	22 924	37 254
LAI	14.51	14.32	13.65	12.04	12.9	15.15	12.19	14.56
BUSCO (%)	95.30	95.00	96.10	93.70	94.10	95.20	95.60	96.40

To facilitate the gene annotation of the assembled 821 genome, we exploited a total of ~46 Gb Iso-seq of various tissues from 821, the previously released Illumina RNA-seq data of Harukei-3 [6], and RNA-seq of nine tissues from the National Center for Biotechnology Information (NCBI), to predict protein-coding genes and establish the gene expression pattern. In total, we obtained 27 685 and 27 258 protein-coding genes of the 821_Hap1 and the 821_Hap2 subgenomes, respectively, using the C&RAP annotation software (Table S2, see online supplementary material) [34]. In addition, ~88.37% (24466) genes of the 821_Hap1 subgenome and ~83.37% (22725) genes of the 821_Hap2 subgenome were supported by multiple functional databases (Fig. S3, see online supplementary material). Moreover, the protein-coding genes were unevenly distributed along each chromosome with higher gene density in both distal regions (Fig. 1C). The estimation of the Benchmarking Universal Single-Copy Orthologs (BUSCO) gene set [35, 36] showed that 95.70% (821_Hap1) and 92.00% (821_Hap2) of the core eudicots genes were detected in the 821 genome annotated gene set. This was higher than the value obtained in other melon genome assemblies, indicating the great accuracy and completeness of the 821 genome assembly and gene models (Table S2, see online supplementary material). Taken together, our results demonstrated that we achieved a haplotype-resolved and chromosome-level genome of 821 (Fig. 1C).

### Consistency and quality of the 821 assembly

**Figure 2:**
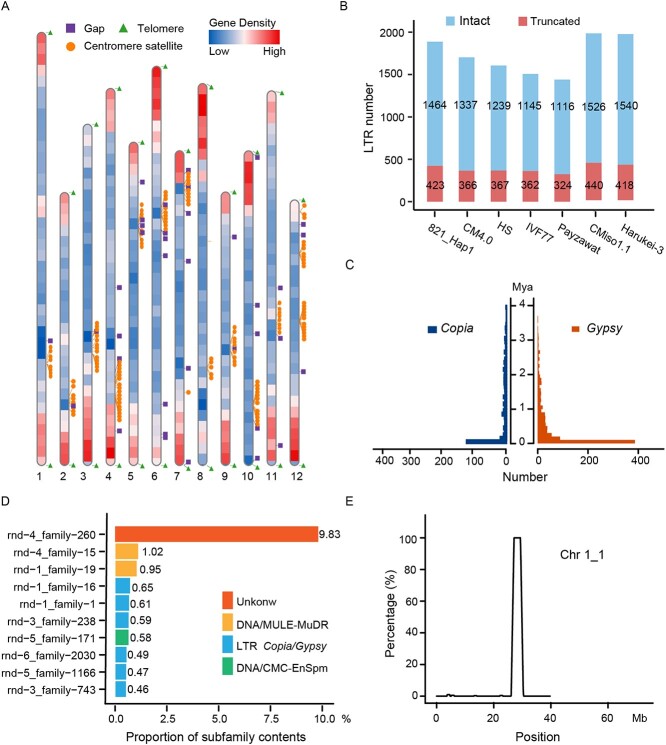
Repetitive sequence analysis of 821. **A** Telomeres, centromere satellites, and gaps distribution on each chromosome of the 821_Hap1 subgenome. The color intensity represents the gene density (number of genes per Mb). Square, triangle, and circle markers represent the positions of gaps, telomeres, and centromeres, respectively. **B** The LTRs statistics of seven melon genomes. **C** The estimated insertion time of *Gypsy*-type and *Copia*-type LTRs. **D** Proportion of the top 10 TE subfamilies. **E** The frequency distribution of the Unknown-type rnd-4_family-260 subfamily enriched towards the centromere of chromosome 1.

To evaluate the consistency and quality of the 821 assembly, we aligned all available primary data, including Illumina reads for survey analysis, HiFi reads for contig assembly, and Hi-C reads for contig anchoring and ordering, to the 821 genome assembly to identify both small and structural variants. We corrected 26 errors in the genome assembly and identified 1779 syntenic blocks and 570 heterozygous variants between the 821_Hap1 and 821_Hap2 subgenomes (Fig. S4, see online supplementary material). Moreover, we detected 23 218 homologous gene pairs between the two haplotypes. Using the 7-bp conservative telomere sequences, we further identified 24 telomeres on 12 chromosomes of 821_Hap1 and 22 telomeres on 12 chromosomes of 821_Hap2 subgenomes, indicating a near complete telomere-to-telomere assembly of the 821 genome (Fig. 2A; Table S3, see online supplementary material). Furthermore, we also identified all centromeric regions harboring centromere satellite arrays on each chromosome, although chromosome 12 of the 821_Hap1 subgenome contained three potential regions (Fig. 2A; Table S3, see online supplementary material). Mapped sequencing reads showed uniform coverage across all chromosomes, with more than 99.50% of the assembly mapped by Illumina and HiFi reads. These data confirmed the high accuracy and consistency of the T2T-821 genome assembly.

To estimate the k-mer-based (k = 19 bp) quality of the T2T-821 genome assembly, we used Merqury [37] with Illumina reads and the results showed that the Merqury estimated QV values were 47.62 (821_Hap1) and 45.86 (821_Hap2), respectively. Furthermore, we assessed the LTR assembly index (LAI) [38, 39] for seven melon genomes, and found that the LAI values for the T2T-821 genome assembly were 14.51 (821_Hap1) and 14.32 (821_Hap2), higher than the reference genome CM4.0 (13.65) value (Table 1). Additionally, we calculated the GC content and depth distribution for the T2T-821 genome using HiFi reads and found no sequencing contamination (Fig. S5, see online supplementary material). These results further confirmed the high quality of the T2T-821 genome.

Due to the high sequence similarity between both haplotypes of the T2T-821 genome, we used the 821_Hap1 subgenome for further analysis. The 821_Hap1 subgenome and the CM4.0 genome were comparatively collinear, with 5959 syntenic blocks containing 22 790 and 24 390 genes, respectively (Fig. S6 and Table S4, see online supplementary material). Furthermore, syntenic analysis between the 821_Hap1 subgenome and other melon genomes showed a higher degree of collinearity (Table S4, see online supplementary material). We also detected several large structural variations between the CM4.0 and the two subgenomes, including five inversions on chromosome 6 (Fig. S6, see online supplementary material), which were consistent with previously reported studies [5, 8]. To validate the accuracy of these inversions, we checked the continuous interaction signals on the Hi-C heatmap (Fig. S7, see online supplementary material) and confirmed that they had not been misassembled, which further proved the correctness of the T2T-821 genome assembly.

### Transposable element analysis

A total of 250.66 Mb of repetitive sequences were identified, accounting for 61.85% of the 821_Hap1 subgenome, which was higher than the values reported in other melon genomes (Table S5, see online supplementary material). Among these repetitive sequences, the long terminal repeat retrotransposons (LTRs) were the predominant repeats covering ~25.52% (103.43 Mb) of the 821_Hap1 subgenome, and the *Gypsy* and *Copia*-type LTRs were the largest LTR subfamilies, accounting for 12.89% (52.26 Mb) and 9.50% (38.51 Mb), respectively (Table S6, see online supplementary material). We further compared the intact and truncated LTRs in the seven genomes and found 1887 LTRs in the 821_Hap1 subgenome, higher than most of the other genomes, but slightly lower than the CMiso1.1 and Harukei-3 genomes, which were assembled using other assembly technologies (Fig. 2B). To estimate transposon activity, we identified a total of 1464 high-confidence full-length LTRs in the 821_Hap1 subgenome (Fig. 2B). The *Gypsy* and *Copia*-type LTRs, especially the former, showed a recent insertion burst around 1 Mya (Fig. 2C). In addition, we identified the top 10 TE subfamilies (greater than 0.4% of the assembled genome length), which included six *Copia*/*Gypsy* subfamilies, three MULE-MuDR/CMC-EnSpm subfamilies and one Unknown subfamily. Together, these subfamilies accounted for approximately 15.65% (~64.24 Mb) of the assembled 821_Hap1 subgenome (Fig. 2D). Only the unknown rnd-4_family-260 subfamily was enriched towards the centromeres of each chromosome of the 821_Hap1 subgenome but absent from the rest of the assembled genome (Fig. 2D and E; Fig. S8, see online supplementary material). These results further confirmed that a burst of LTRs was the major driving force for the expansion of the melon genome [40].

### Structural variations between the *Melo* and *agrestis* genomes

**Figure 3:**
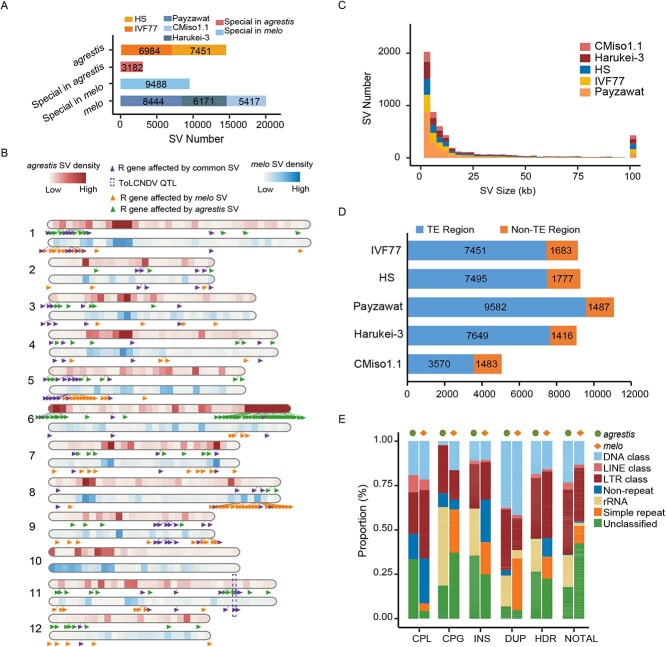
Structural variations between *melo* and *agrestis*. **A** The number of structural variations between *melo* and *agrestis*. **B** The distribution of *melo* and *agrestis* SVs in the 12 chromosomes and resistance genes affected by *melo* and *agrestis* SVs, respectively. **C** The length distribution of SVs among five melon genomes. **D** The number of SVs located in TE regions. **E** The sequence composition of *melo* and *agrestis* SVs. CPL, copy loss; CPG, copy gain; DUP, duplication; HDR, highly diverged regions; INS, insertion; NOTAL, un-aligned region.

Structural variations (SVs) play important roles in the formation of plant characteristics during domestication. The two subspecies *melo* and *agrestis* have been domesticated independently and cultivated in different geographical areas, which resulted in diverse phenotypic characteristics and adaptation [2]. To elucidate the genetic basis underlying these divergences, we aligned three *melo* (CMiso1.1, Harukei-3, Payzawat) and two *agrestis* genomes (HS, IVF77) to the 821_Hap1 subgenome to identify genetic variations. We revealed a total of 20 032 (~141.98 Mb in total) and 14 435 (~124.65 Mb in total) SVs with the *melo* and *agrestis* genomes, respectively (Fig. 3A). These SVs were unevenly distributed on all chromosomes (Fig. 3B). Nearly 10.16% of SVs were larger than 5 Kb, with 74.08% being <500 bp (Fig. 3C). Further analysis demonstrated that most of the SVs identified in each genome were located in TE regions (Fig. 3D;Fig. S9, see online supplementary material). The proportion of TE families showed that several TEs contained in the SVs had significant divergence within the *melo* and *agrestis* genomes, such as the Simple repeat and rRNA families (Fig. 3E). Among all these SVs, we identified 3182 (~73.48 Mb) *agrestis*-specific SVs and 9488 (~61.62 Mb) *melo*-specific SVs (Fig. 3A). Furthermore, we detected that 1067 *agrestis*-specific SVs and 1514 *melo*-specific SVs resided in *agrestis* and *melo* sweeps, respectively, of which 25 *agrestis*-specific SVs and 548 *melo*-specific SVs overlapped with QTLs and genes previous reported (Table S7, see online supplementary material) [2]. Of these, ten QTLs and one gene associated with fruit length, fruit weight, flesh thickness, fruit diameter, and fruit bitterness reported previously [2], were located in *melo*-specific SVs, and four QTLs resided in *agrestis*-specific SVs. Furthermore, we found that SVs within the *melo* domestication sweep ‘WM143’ containing the *CmBi* gene encoding a cucurbitadienol synthase were highly diverse in the *agrestis* genome, whereas this region was fixed in the *melo* genome (Fig. S10, see online supplementary material), which was consistent with previous results [2]. These results suggest that *CmBi* was domesticated in *melo* and further support the conclusion that independent domestication events took place in melon.

Disease resistance in plants is frequently associated with SV in the form of tandem arrays of resistance genes [41, 42]. To investigate resistance gene analogs between the *melo* and *agrestis* genomes reported previously, we performed a homolog analysis of resistance (R) genes, and identified 1028 *R* genes in the 821_Hap1 subgenome (Fig. S11, see online supplementary material), of which 238 (23.15%) were significantly overlapping with SVs (Fig. 3B). Among these genes, we discovered that 105 and 87 were affected by *agrestis* and *melo*-specific SVs, respectively. Additionally, the *R* genes were unevenly distributed on each chromosome (Fig. S11, see online supplementary material). We found that 42 (85.71%) *R* genes affected by the *agrestis*-specific SVs were enriched on chromosome 6, and 21 (77.78%) *R* genes affected by the *melo*-specific SVs were enriched on chromosome 8 (Fig. 3B). Interestingly, no *R* genes affected by SV were detected on chromosome 10 in the *melo* and *agrestis* genomes. These results indicate that most *R* genes might have been independently selected during *melo* and *agrestis* domestication.

### Functional impact of one plausible CNV to ToLCNDV resistance

**Figure 4:**
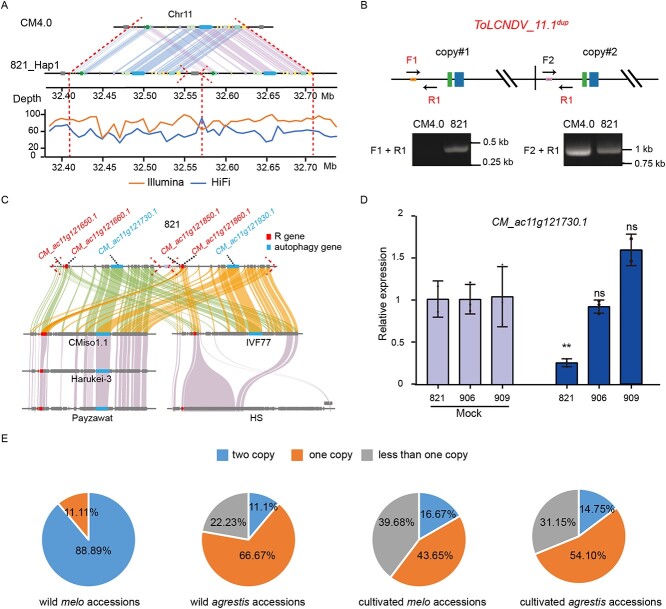
The *ToLCNDV_1.1^dup^* associated with ToLCNDV resistance. **A** The copy number variation region in chromosome 11. **B** The PCR validation of the *ToLCNDV_1.1^dup^*. **C** Gene collinearity analysis of *ToLCNDV_1.1^dup^* within six genomes. **D** Relative expression level of *CM_ac11g121730.1* in different melon accessions measured by qRT-PCR. The values are presented as the mean ± SD (n = 3 biological and 3 technical replicates). **P < 0.01 (Student’s *t*-test). **E** The proportion of the *ToLCNDV_11.1^dup^* existing in the wild and cultivated accessions.

ToLCNDV was first reported in India and rapidly spread to Mediterranean and Asian countries, such as Spain, Pakistan, Thailand, Iran, and Indonesia [43–47]. Recently, ToLCNDV has been observed in China, indicating that the virus will become a new threat to numerous vegetable crops [48]. Numerous reports have shown ToLCNDV as the first DNA bipartite begomovirus (*Geminiviridae*), causing severe yield and economic losses in cucurbit crops [49]. The melon accession 821 possesses high resistance to ToLCNDV [16, 17]. A previous study identified several QTLs and candidate genes associated with resistance to ToLCNDV [49]. The homology analysis showed that three loci associated with ToLCNDV resistance were detected in the 821_Hap1 subgenome. A major ToLCNDV resistance QTL harboring one R gene was reported to reside in chromosome 11 [50]; however, we found eight R genes of the 821_Hap1 subgenome in this QTL. Sequence analysis discovered a copy number variation (CNV) in the 821_Hap1 subgenome, and alignment analysis showed abnormal read pairs and read depths, which strongly supported this CNV (Fig. 4A). Furthermore, the detection of copy number by PCR and qRT-PCR supported that the CNV (designated *ToLCNDV_11.1^dup^*) was present twice in the 821_Hap1 subgenome (Fig. 4B; Table S8, see online supplementary material). Intriguingly, the *ToLCNDV_11.1^dup^* region contains four R genes harboring the TM-Kinase domain, *CM_ac11g121650.1* and *CM_ac11g121850.1*, encoding a peptidyl tyrosine phosphorylation protein, and *CM_ac11g121660.1* and *CM_ac11g121860.1*, encoding a phosphorylation protein (Fig. 4C). However, in a previous study differential expression levels of the four R genes were not observed after artificial inoculation with ToLCNDV on resistant and susceptible melon accessions (Fig. S12, see online supplementary material) [17]. Autophagy is also involved in programmed cell death and disease resistance in plants [51]. Coincidentally, we also identified two genes (*CM_ac11g121730.1* and *CM_ac11g121930.1*) in *ToLCNDV_11.1^dup^* in the 821_Hap1 subgenome, involved in the plant autophagy pathway and encoding autophagy protein six. The expression levels of *CM_ac11g121730.1* in resistant accessions were significantly lower than that in susceptible accessions after artificial inoculation (Fig. 4D), indicating that it played a negative role in resistance to ToLCNDV, which was consistent with a previously report in Arabidopsis [51]. Further, the autophagy genes could mediate the degradation of transcriptional activators to enhance ToLCNDV resistance in tomato [52]. Therefore, we speculate that the autophagy process could play important roles in ToLCNDV resistance.

We further compared this *ToLCNDV_11.1^dup^* with its homologous regions in the *melo* and *agrestis* genomes and discovered one copy of *ToLCNDV_11.1^dup^* present in three *melo* genomes (Fig. 4D). To dissect the distribution of the *ToLCNDV_11.1^dup^* in melon, we checked 206 wild and cultivated accessions of *melo* and *agrestis* subspecies from a previous study [2], and found that 88.89% (8) wild *melo* accessions and 11.11% (1) wild *agrestis* accessions possessed the duplication (Fig. 4E), indicating that the *ToLCNDV_11.1^dup^* might be diversified within the wild melon. Additionally, the loss of the *ToLCNDV_11.1^dup^* was more significant in the *melo* subspecies (Fig. 4E), suggesting that the ToLCNDV resistance might have experienced independent selection during *melo* and *agrestis* subspecies domestication. However, the association of *ToLCNDV_11.1^dup^* with ToLCNDV resistance needs to be further verified in future experiments.

### Phenotypic impact of SNPs related to fruit ripening

**Figure 5:**
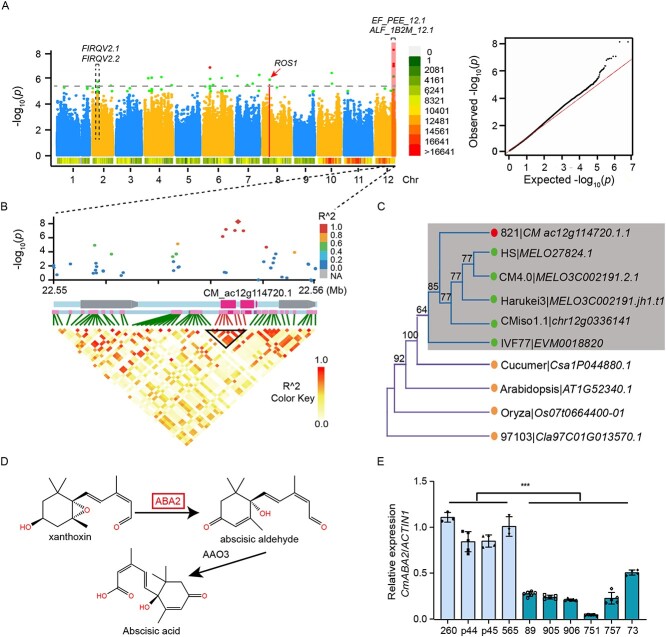
Identification of a candidate gene for the melon climacteric trait. **A** Manhattan plot of GWAS on the climacteric trait across all chromosomes. Black bars represent previous QTLs results and genes associated with climacteric traits. **B** Local Manhattan plot (top), genes in LD block (middle), and a representation of the pairwise R^2^ values (bottom) surrounding the peak on chromosome 12. **C** Phylogenetic tree of the *CmABA2* protein in melon and its homologs in other species. **D** The last two steps of the ABA biosynthesis pathway. **E** Relative expression level of the *CmABA2* in four non-climacteric and six climacteric accessions. The values are presented as the mean ± SD (*n* = 3 biological and three technical replicates). ***P < 0.001 (Student’s *t*-test). The 260, P44, p45 and 565 are climacteric accessions and 89, 905, 906, 751, 757, and 73 are non-climacteric accessions.

Climacteric respiration plays an important role in the ripeness of melon. Melon is a unique model species for studying fruit ripening because of presenting both climacteric and non-climacteric types. Recently, a few QTLs, *ETHQV8.1*, *ETHQB3.5* and *ETHQV6.3* were detected, and several candidate genes were confirmed to be associated with ethylene biosynthesis regulating fruit ripening and climacteric respiration [18–22]. In general, climacteric respiration is closely related to aroma formation. Two QTLs (*EF_PEE_12.1*, *ALF_1B2M_12.1*) for fruit aroma biosynthesis influencing the synthesis of 2-methylbutanol and ethyl propanoate, respectively, have been identified on chromosome 12 [53]. We investigated 117 climacteric and 82 non-climacteric melon accessions (Table S9, see online supplementary material) and downloaded their whole genome re-sequencing data from a previous study [2]. Interestingly, we identified a strong GWAS association signal for the climacteric trait (*P* = 9.34 × 10^−8^) on chromosome 12 within an interval of 0.66 Kb based on the 821_Hap1 subgenome (Fig. 5A). In addition, several previously reported QTLs and genes associated to fruit ripening and aroma biosynthesis [53] overlapped with significant signals (Fig. 5A). The LD analysis showed that ten SNPs formed a 1.7 Kb linked interval, which was located in the gene *CM_ac12g114720.1* (*CmABA2*) encoding a cytoplasmic short-chain dehydrogenase/reductase (Fig. 5B). Cytoplasmic short-chain dehydrogenase/reductase could play a critical role in regulating the anthoxin content during ABA biosynthesis [54]. The *ABA2* orthologs are conserved among species and have been confirmed to be involved in the conversion of xanthoxin to ABA-aldehyde in the ABA biosynthesis pathways in rice and *Arabidopsis* (Fig. 5C and D).

Previous studies have demonstrated that ABA biosynthesis precedes ethylene and promotes ethylene biosynthesis during fruit ripening [55–57]. Among the significant SNPs (−log_10_(*P*) ≥ 5.6), we identified a nonsynonymous mutation in the *CmABA2* gene, resulting in a phenylalanine change to isoleucine. The qRT-PCR experiment revealed that the expression levels of *CmABA2* in four non-climacteric accessions (260, p44, p45, 565) were significantly higher than that in six climacteric accessions (89, 905, 906, 751, 757, 73) in mature fruits (Fig. 5E). These data suggest that *CmABA2* might play an important role in non-climacteric fruit ripening.

## Discussion

A semi-wild accession, 821, belongs to *C. melo ssp. agrestis* var. *acidulus*. Previous studies revealed that accessions from *C. melo ssp. agrestis* var. *acidulus* are resistant to a variety of pests and virus diseases [15, 31, 58, 59]. As a representing accession in the *C. melo ssp. agrestis* var. *acidulous*, 821 was demonstrated to be resistant to powdery mildew and to a variety of viruses transmitted by whiteflies, such as CYSDV, CABYV, WmCSV, LIYV, CuLCrV, and ToLCNDV [16, 31, 60]. In addition, the fruit of 821 is non-climacteric and has a long shelf life after harvest. This indicates that 821 may contain genes associated with disease resistance and extended shelf life of fruit. Therefore, the high-quality genome assembly of 821 is a valuable resource for the identification of candidate genes associated with important agronomic traits in melon.

In this study, we assembled two haplotypes of the semi-wild melon of 821, including a 373.31-Mb 821_Hap1 subgenome containing 27 685 genes and a 364.05-Mb subgenome containing 27 258 genes. Their contig N50 sizes were 10.26 and 11.86 Mb, respectively, which was higher than that of the previously reported melon genomes (Table 1). The telomeres, centromeres and gaps of the 821 genome suggested the complete T2T structure in all 12 chromosomes of the 821_Hap1 subgenome (Fig. 2A; Table S3, see online supplementary material). A total of 2103 protein-coding genes were found in the centromeric region of the two haplotypes. A functional enrichment analysis showed that these genes were involved in ovule, carpel, and gynoecium development (Fig. S13, see online supplementary material). Gene collinearity analysis found that 6028 genes in the 821 genomes had low similarity in the CM4.0 genome. The GO analysis revealed that these genes were related to the TOR signaling pathway, pollen tube tip development and pollen-pistil interaction (Fig. S14, see online supplementary material). The gaps within the T2T-821 genome were reduced to 38.50 Kb, accounting for 0.005% of the genome (Table S10, see online supplementary material), and the integrity of the T2T-821 genome was higher compared with that of previous melon genomes. However, the complete gap-free assembly of the melon genome will require additional efforts.

During melon domestication, *melo* and *agrestis* were domesticated independently, which could leave imprinting in their genomes [2]. However, a diverse comparative genomic analysis based on genomic sequences between *melo* and *agrestis* has not been reported. In this study, multiple melon genomes were explored to identify the *melo* and *agrestis*-specific SVs. The differences in the TE composition of SV between *melo* and *agrestis* genomes suggested that TEs might have been subjected to differential selection for independent domestication events. Furthermore, we also compared the *R* genes affected by SV, and found significant differences on chromosomes 6, 8, and 10, which revealed that *R* genes might have undergone differential selection during independent domestication.

ToLCNDV is a whitefly-transmitted plant virus that has been affecting European melon cultivations for over a decade, which spread to China recently [45, 48, 61]. It has been considered to be a great threat to melon production worldwide. Several QTLs for ToLCNDV resistance have been reported in cucurbits [50, 59, 62]. In melon, a major dominant locus of ToLCNDV resistance was mapped on chromosome 11, and two genes encoding a TIFY4B transcription factor and a serine/threonine-protein kinase PBS1 were considered as the candidate genes [50]. Intriguingly, eight R genes were predicted in the major QTL for ToLCNDV resistance on chromosome 11 in the T2T-821 genome, but only one gene was detected in the previous study [50]. However, we did not detect differences in the expression levels of these R genes after artificial inoculation with ToLCNDV to resistant and susceptible melon germplasm reported in a previous study (Fig. S12, see online supplementary material) [63]. Furthermore, previous research indicated that the ToLCNDV transcription activator protein could be degraded through the autophagy pathway [52]. The autophagy pathway was found to respond to plant disease resistant negatively in Arabidopsis, supporting that autophagy genes in the *ToLCNDV_11.1^dup^* might play a role in resistance to ToLCNDV infection, but additional experiments are still needed. The discovery of *ToLCNDV_11.1^dup^* is attributed to the continuity and integrity of the melon genome of the resistant accession 821 [17]. This suggests that it is important to assemble the genome of wild or semi-wild accessions for identifying resistant genes. The T2T-821 genome will provide great potential for melon resistance breeding as it is resistant to multiple diseases.

Climacteric ripening is an important agronomic character directly connected to fruit ripening, aroma formation, and shelf life. Fruit ripening is a complex regulatory process, which is mainly controlled by phytohormones, including abscisic acid (ABA), ethylene, and auxin [64–66]. Ripening of climacteric fruits is dependent on the ethylene production, while the ripening of non-climacteric fruits is controlled by ABA [27, 66]. Ripening of fleshy fruits, such as tomato, depends on the synergistic effect of ethylene and ABA [27, 67]. However, previous studies on melon fruit ripening mainly focused on ethylene biosynthesis and its regulation [19–21, 23]. However, ABA and its interaction with ethylene during melon fruit ripening still remain un-elucidated. During the ripening process, ABA could accumulate at the beginning of ripening [68], and further promote the biosynthesis of ethylene as a signaling molecule [56, 69]. Additionally, some transcription factors involved in ethylene synthesis are regulated by ABA [69]. In this study, we performed a genome-wide association study (GWAS) analysis and identified one gene, *CmABA2,* involved in the conversion of xanthoxin to ABA-aldehyde during ABA biosynthesis. The expression level of *CmABA2* in non-climacteric melon accessions is higher than that in climacteric ones, which indicates that *CmABA2* may play an important role in fruit ripening. Therefore, the T2T-821 genome availability may be helpful to identify candidate genes and variations related to climacteric ripening, and develop varieties with long shelf life.

In summary, the T2T-821 genome of melon was assembled with high reliability and quality. Novel genetic variations between *melo* and *agrestis* were identified containing a CNV (*ToLCNDV_11.1^dup^*) that may have an impact on ToLCNDV resistance. In addition, a major locus controlling fruit climacteric during ripening related to ABA regulation has been identified. The results of this study will provide potential value and information for melon breeding and the understanding of the domestication process.

## Materials and methods

### Plant materials and sequencing

The 821 accession was self-fertilized from PI 313970 (*C. melo ssp. agrestis* var. *acidulus*) for several generations, and planted in the Zhengzhou Fruit Research Institute, Chinese Academy of Agricultural Sciences (ZFRI, CAAS). The leaves of 821 were used to construct the PacBio SMRT library for genome sequencing. The Hi-C libraries were built according to the Proximo Hi-C plant protocol with the restriction enzyme *Dpn*II. The mixed tissues of the 821 variety were used to construct a PacBio Iso-Seq library and then sequenced. The 200 climacteric and non-climacteric melon accessions were selected from a previous study [2].

### Genome assembly and quality evaluation

The 821 genome was assembled using HiFi data from PacBio circular consensus sequencing technology associated with the Hi-C method. First, the 821_Hap1 and 821_Hap2 subgenomes were assembled under the Hi-C mode of Hifiams using HiFi data (V0.16.1-r375) [33] with the default parameters. The 821 genome continuity was evaluated by the contig N50 length. The Hi-C data was aligned to the 821_Hap1 and 821_Hap2 subgenomes, respectively, and classified as valid or invalid interaction pairs using the Juicer pipeline [70]. Meanwhile, misassembled contigs were detected through Juicebox (V1.11.08) [71] and 3D-DNA pipelines (V180114) [72] and corrected manually. After adjusting the assembly errors of the 821_Hap1 and 821_Hap2 subgenomes, purge_haplotigs [73] was used to evaluate whether the chimeras were cleared. In addition, we calculated the heterozygosity through GenomeScope2 [74] with 19-mers.

The HiFi data was mapped to the 821 genome with minimap2 [75] and the sequencing depth and coverage across the whole genome were calculated. The completeness of the assembled 821 genome was evaluated by BUSCO (V4.1.4) [35, 36] using the embryophyta_odb10 database. We also assessed the assembly quality of the 821 genome using the Merqury [37] quality value (QV) based on the 19-mer. Minimap2 [75] and MUMmer package (V3.23) [76] were used to perform a genome-wide comparison between the 821_Hap1 and 821_Hap2 subgenomes. The distribution of centromeres, telomeres, and gaps on each chromosome of 821_Hap1 and 821_Hap2 subgenomes was counted and mapped by the RIdeogram [77].

### Repeat analysis and gene annotation

A repeat library of the 821 genome was built using Repeat-Modeler (V2.0.1) [78] and LTR_retriever (V2.9.0) [39], which was further used to identify TEs with RepeatMasker (V4.1.0) [79]. The LAI value [38] was used to evaluate the seven melon genomes. The telomeres of the 821 genome were identified using Tandem Repeat Finder (TRF) [80]. A candidate subfamily of centromere tandem repeats was identified through estimating the frequency distribution of the rnd-4_family-260 subfamily across the whole genome.

The transcriptome from the Iso-seq platform was processed using the Iso-Seq pipeline (V3.1) to produce complete mRNA sequences [81]. Both transcriptome evidence from the Iso-seq and Illumina platforms were mapped to the 821 genome to predict protein coding genes using combining and comparing annotation methods, C&RAP (V1.0) [34], through AUGUSTUS (V3.4.0) [82], MAKER2 [83], and GeMoMa (V1.7.1) [84]. Meanwhile, the output of prediction results from three platforms were combined and compared by C&RAP (V1.0) and were modified through manual correction. The gene completeness was evaluated by BUSCO (V4.1.4) [35, 36] using the embryophyta_odb10 database. NR, Swiss-Prot, and the *Arabidopsis* database were used to predict the functions of 821 protein-coding genes using Diamond (V0.9.24.125) [85]. The InterProScan [86] and KEGG Automatic Annotation Server [87] were used to preform protein domain and gene ontology term annotations. We used DRAGO3, a tool of PRGdb (V4.0) [88], to predict R genes of the 821_Hap1 subgenome, and R packages (V4.1.3) was used to map the distribution of R genes on each chromosome of the 821_Hap1 subgenome.

### Genome wide comparison of melon genomes

The genome wide comparisons between the 821_Hap1 subgenome and other melon genomes were performed through minimap2 [75] and MUMmer package (V3.23) [76]. The Syri (V1.5.4) [89] was performed to identify structure variants between seven melon genomes. Firstly, we excluded structural variants of less than 50 bp, and secondly selected structural variants in the same region on the 821_Hap1 subgenome. Then the SnpEff [90] software was used to annotate the structural variants. Genes located at the region of SVs were considered to be affected. The sequence type and repeat sequence content statistics of SVs were calculated using self-script. The distribution of SVs and R genes influenced by SVs on each chromosome was mapped by the RIdeogram [77]. For each pairwise alignments, the coding sequences of the predicted genes of the 821_Hap1 subgenome were compared with other melon genomes using all-versus-all BLASTP (e-value <10^−5^). The syntenic blocks analysis between 821 and other melon genomes were performed by MCScanx [91] with the default parameters containing at least five collinear genes.

### Validation of *ToLCNDV_11.1^dup^*

qRT-PCR was used to detect the copy number of *MELO3C022300* and *MELO3C022301* in the 821 variety. *CmACTIN* was used as an internal control and 5 gradient diluted genome DNAs of CM4.0 were used as the template. y = −1.72X + 23.3, R^2^ = 0.90, and the standard curve of the cycle threshold (Ct) relative to the log of each initial template copy of *CmACTIN* were obtained. With the same method, standard curves of the target genes (*MELO3C022300* and *MELO3C022301*) were y = −2.14X + 22.5, R^2^ = 0.95, and y = −2.4X + 21.8, R^2^ = 0.99, separately, which were acquired using 5 gradient diluted DNAs of 821. The Ct values of *CmACTIN* and target genes in DNA samples were obtained by qRT-PCR, and the copy number was calculated by comparing the initial template of the target gene with *CmACTIN*, which was concluded by two qRT-PCR repeats. Three primers were designed to detect the duplication (*ToLCNDV_11.1^dup^*), including the F1 primer sequence 5’-GCAATCAAGCTAAGTAATTAGT-3′, the R1 primer sequence 5’-CAATAACCCATCTTTGTCGAAG-3′ and the F2 primer sequence 5’-GTCGGTACAAATAGGTGTCAG-3′.

### SNP identification and genome-wide association analysis of climacteric fruit

The sequence data of 200 melon climacteric and non-climacteric accessions was mapped to the 821_Hap1 subgenome using BWA (V0.7.17-r1198) [92]. SNPs were called by the HaplotypeCaller module of the GATK toolkit (V3.2–2) [93]. The raw SNPs were acquired with filter expressions (“QD < 2.0 || MQ < 40.0 || FS > 60.0 || MQRankSum < -12.5 || ReadPosRankSum < -8.0” —filterName “SNPsfilter” —clusterSize 3). A total of 2 891 126 SNPs, with MAF >5% and a missing rate <10%, were identified for further analysis. The phenotypes of climacteric and non-climacteric melon accessions were described as 1 and 0, respectively (Table S9, see online supplementary material). The BN (Balding–Nichols) kinship matrix was constructed and the proportion of SNPs was randomly selected with the default parameter (emmax -kin -v -h -d 10) [80]. A *P*-value <0.05 was set for significantly differential level. The effective number of the filtered SNPs was calculated using the Genetic Type Error Calculator software (V0.2; http://grass.cgs.hku.hk/gec/register.php). The Manhattan plot was displayed using R package ‘qqman’ (https://cran.r-project.org/web/packages/qqman/). The function of the *CmABA2* referred to homologous genes in the NCBI database (https://www.ncbi.nlm.nih.gov/).

### Linkage disequilibrium analysis on chromosome 12

To display the pairwise linkage disequilibria between SNPs around the peaks on chromosome 12, the SNP genotypes and physical map were used. The SNPs were filtered in PLINK [94], with parameter —maf 0.05 —geno 0.1. The LD heatmap was constructed using the LDBlockShow (V1.40) [95].

### Construction of the *CmABA2* phylogenetic tree

All the protein sequences of ABA2 orthologs were downloaded from the NCBI database (https://www.ncbi.nlm.nih.gov/) and aligned by ClustalW (V2.1) with default parameters. The maximum likelihood phylogenetic tree of ABA2 was constructed using MEGA (V11) with 1000 repeats of the bootstrap.

### Quantitative real-time PCR

RNA samples of melon seedling leaves were extracted through Trizol reagent (Sigma, Shanghai, China). Reverse transcript PCR (RT-PCR) was carried out using the All-in-one mix kit (Bioman, China). Primers for qRT-PCR were produced by Sangon Biotech (Zhengzhou, China). Relative expression levels of target genes were performed with the SYBR Green FAST Mixture qPCR mix reagent (GenStar, China) using the LightCycler480 (Roche Diagnostics, USA) real-time PCR detection System, and PCR was performed by 95°C for 30 s, 40 cycles of 95°C for 15 s and 60°C for 15 s, and finally 72°C for 30 s. The relative expression levels of the target genes were calculated through the 2^^-ΔΔ Ct^ method.

## Supplementary Material

Web_Material_uhad182

## Data Availability

The original sequencing data for genome assembly have been deposited in the Genome Sequence Archive (GSA) database at BIG Data Center (https://ngdc.cncb.ac.cn/gsa/) with Accession Number CRA010716. The assembled genome 821 was deposited in the Genome Warehouse (GWH) database of the Big Data Center (https://bigd.big.ac.cn/gwh/) under the accession number GWHDOOI00000000. The assembled genome 821 and annotations are available on figshare (10.6084/m9.figshare.23701680).
