# Supplementary material for: The haplotype-resolved T2T reference genome highlights structural variation underlying agronomic traits of melon
Source: Hortic Res. 2023 Aug 28;10(10):uhad182. doi: 10.1093/hr/uhad182 (PMC10599238; doi:10.1093/hr/uhad182)
Supplement: Web_Material_uhad182 [file web_material_uhad182.zip › Figure S1-14_2023.08.24.pdf]

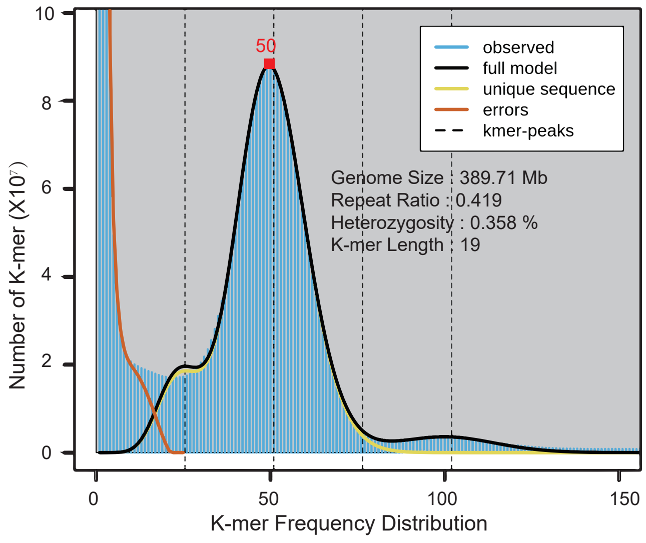

Figure. S1 The distribution of 19-mer in clean sequence data of the 821 genome assembly. The x-axis and y-axis indicate the 19-mer frequency and number, respectively.

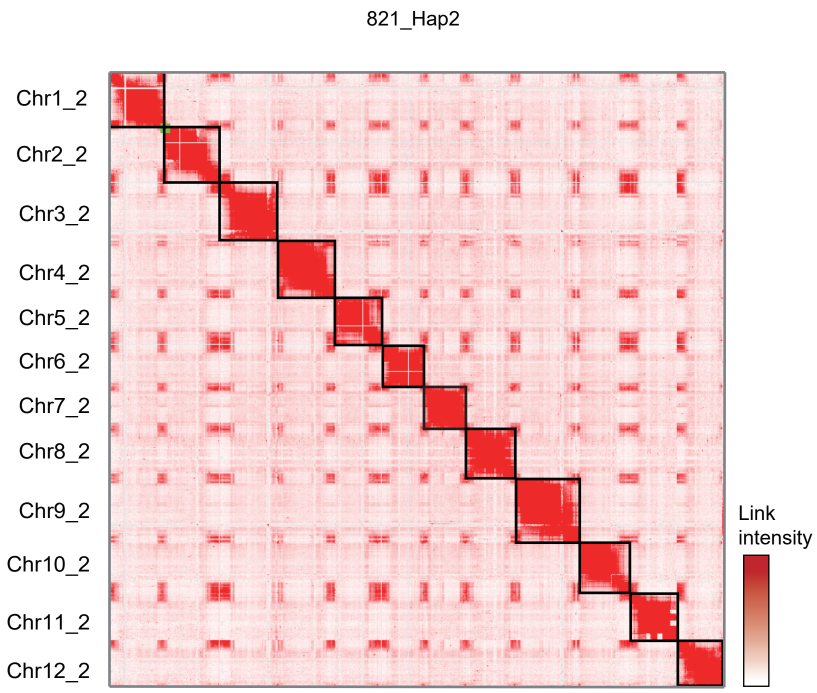

Figure. S2 Hi-C map of the 12 chromosomes of the 821_Hap2 subgenome. Heatmap intensity scale is indicated by the interaction intensity color bar.

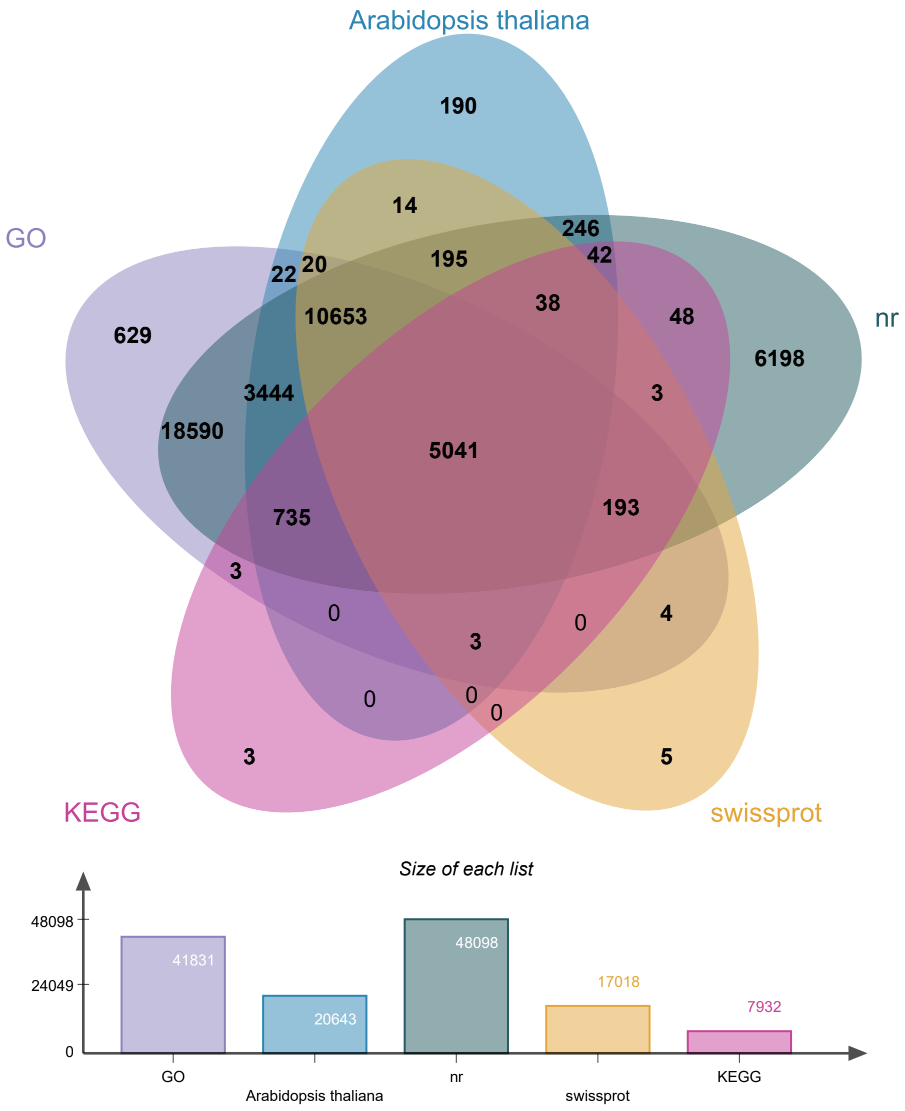

Figure. S3 The numbers of protein-coding genes annotated in the NCBI nr plant, SWISS-PROT, GO, KEGG and Arabidopsis databases are illustrated by Venn diagram.

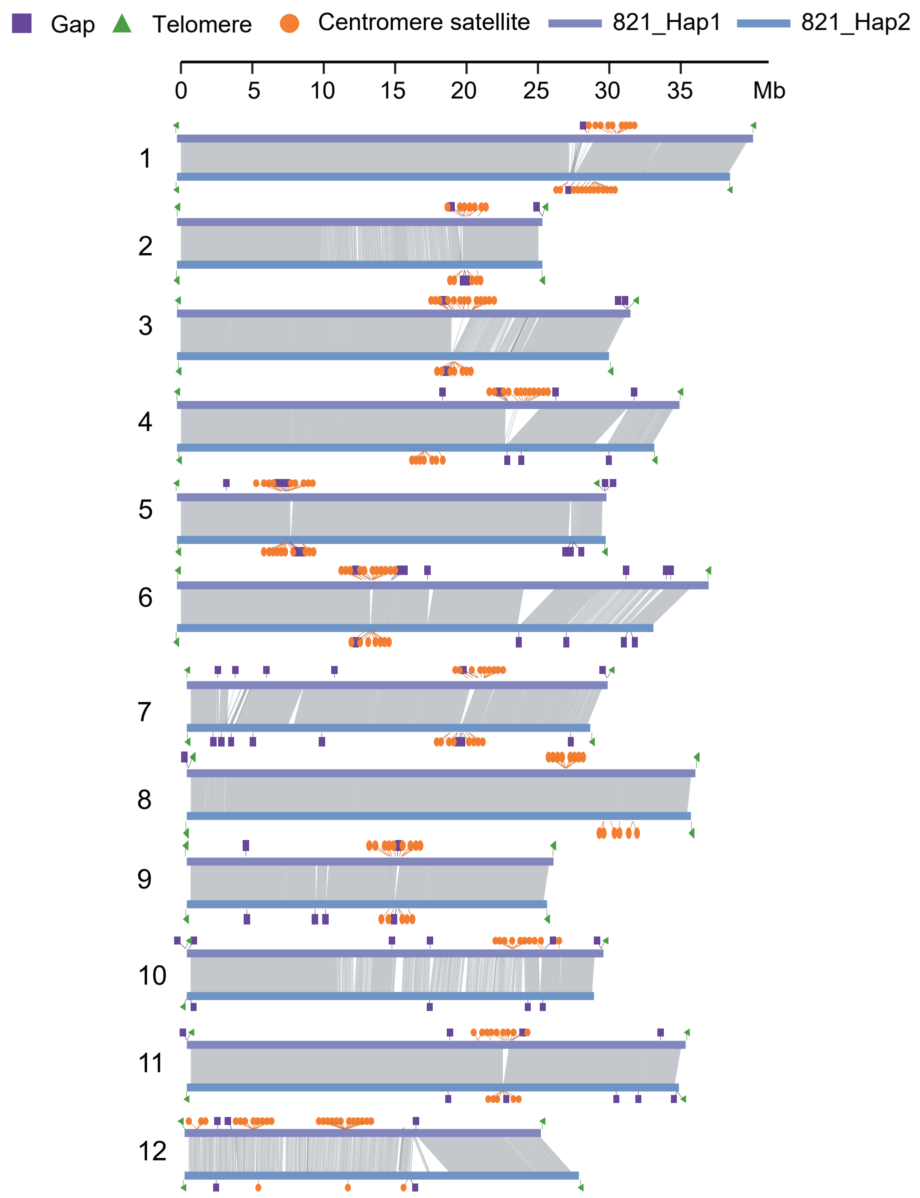

Figure. S4 The collinearity map of the 821_Hap1 and 821_Hap2 subgenome.

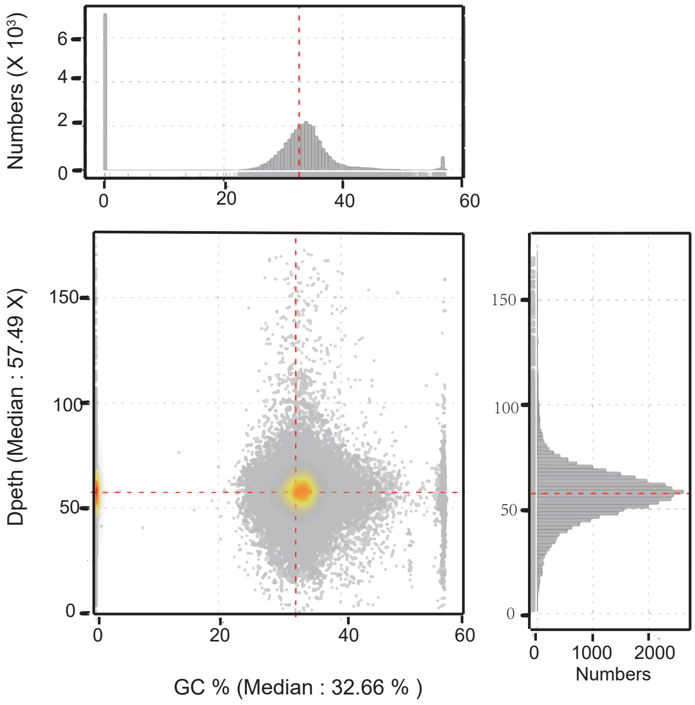

Figure. S5 The GC and depth distribution plot of the 821 genome assembly .
For the scatter plot in the middle, the abscissa is GC content and the ordinate is sequencing depth. For the histogram on both sides, it is the sliding window(10k) frequency distribution of GC content and sequencing depth respectively.
The red dotted line is the median value.

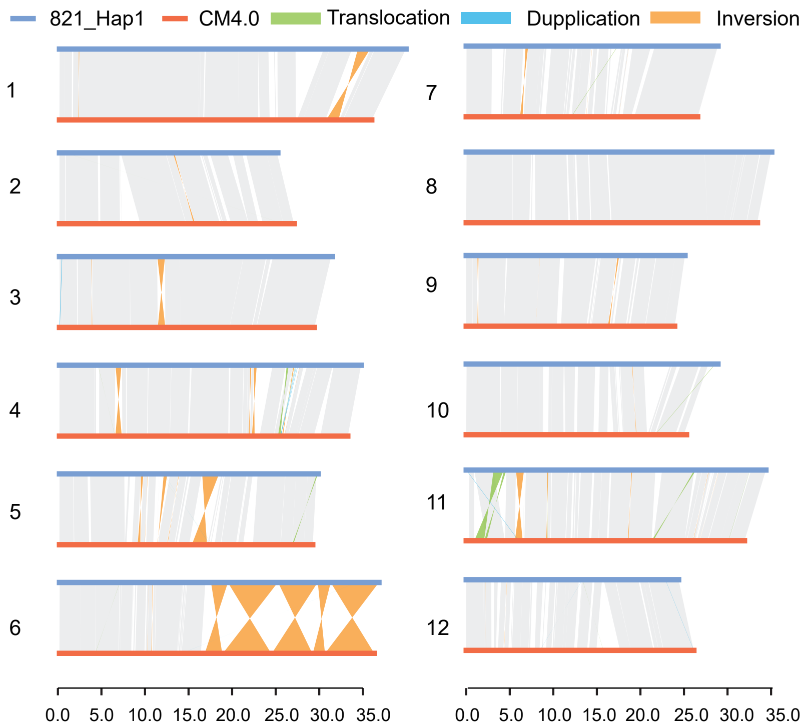

Figure. S6 The collinearity map of the 821_Hap1 subgenome and CM4.0 genome.

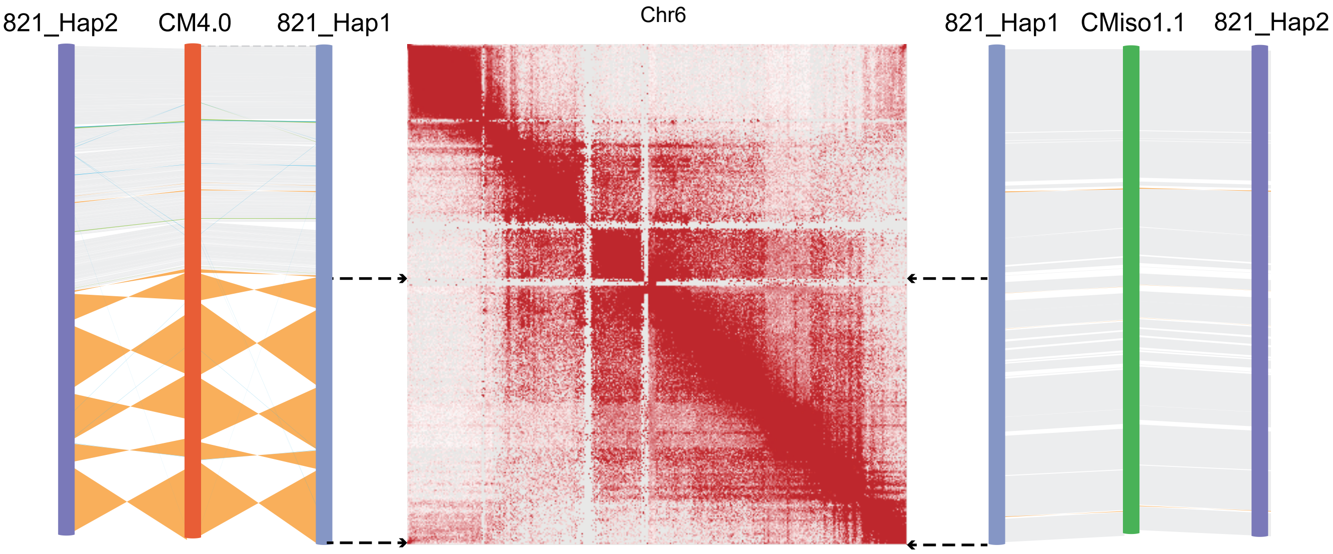

Figure .S7 The collinearity map of chromosome 6 between 821, CM4.0 and CMiso1.1.

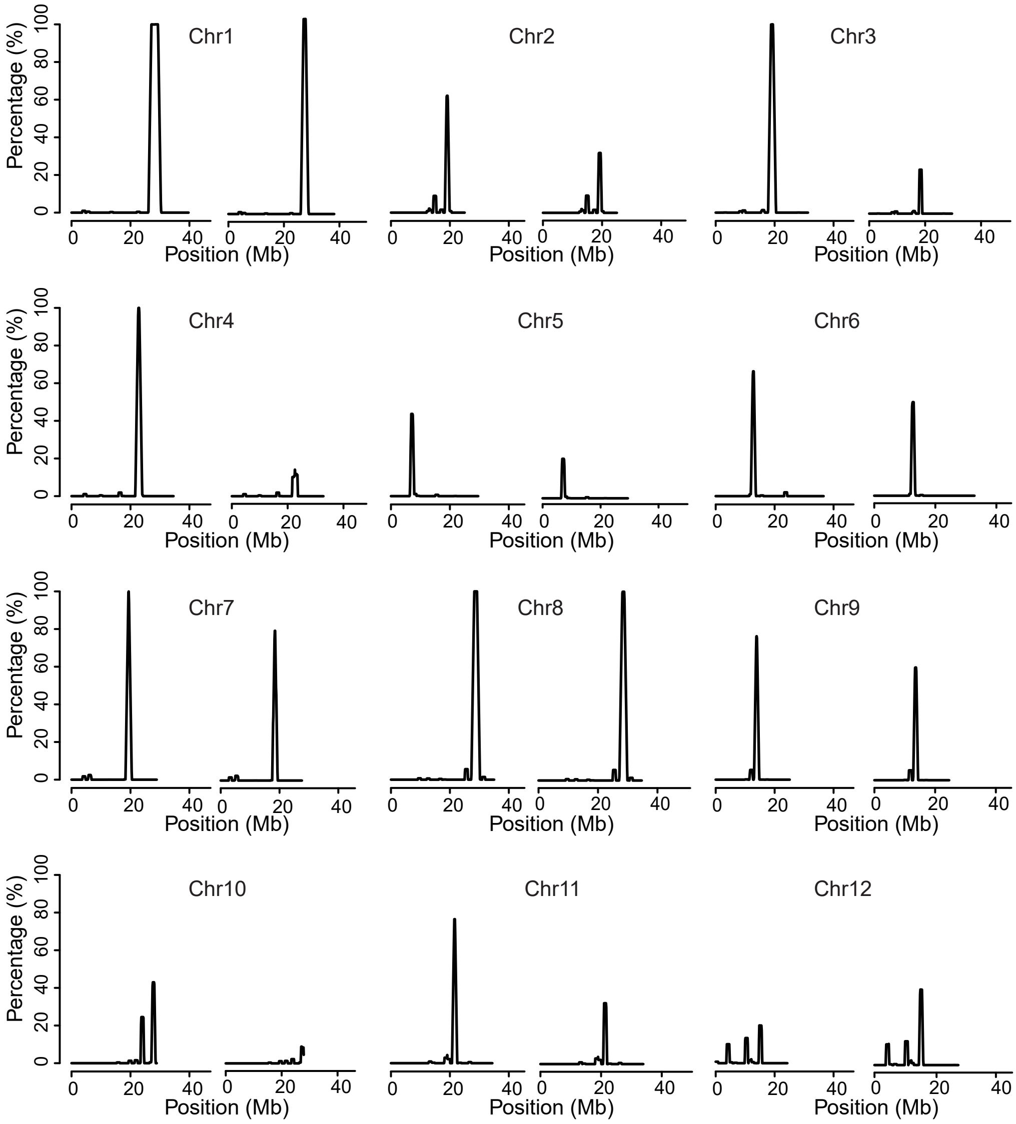

Figure. S8 The frequency distribution of the Unknown-type rnd-4_family-260 subfamily, showing that it was enriched towards the centromere of each chromosome (left: 821_Hap1, right:821_Hap2).

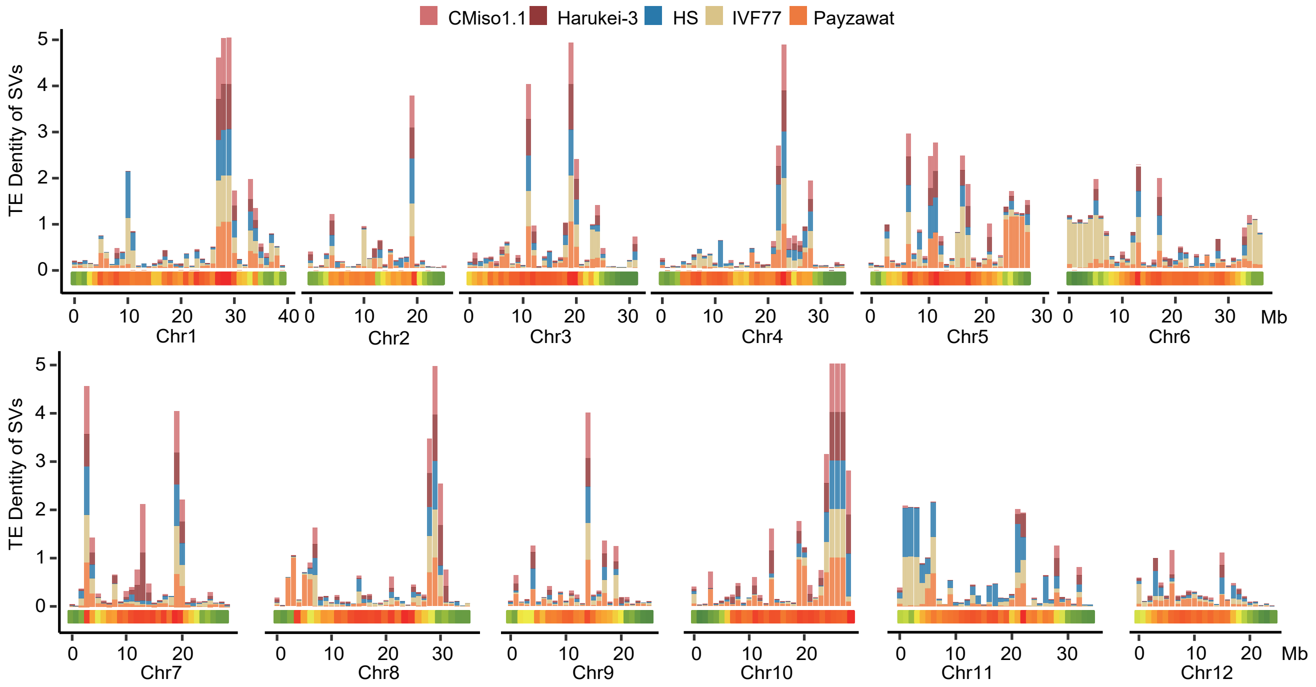

Figure .S9 Genome-wide relationship between TE density and TE content of SVs.The heat map represents TE density and the bar graph represents TE content of SVs.

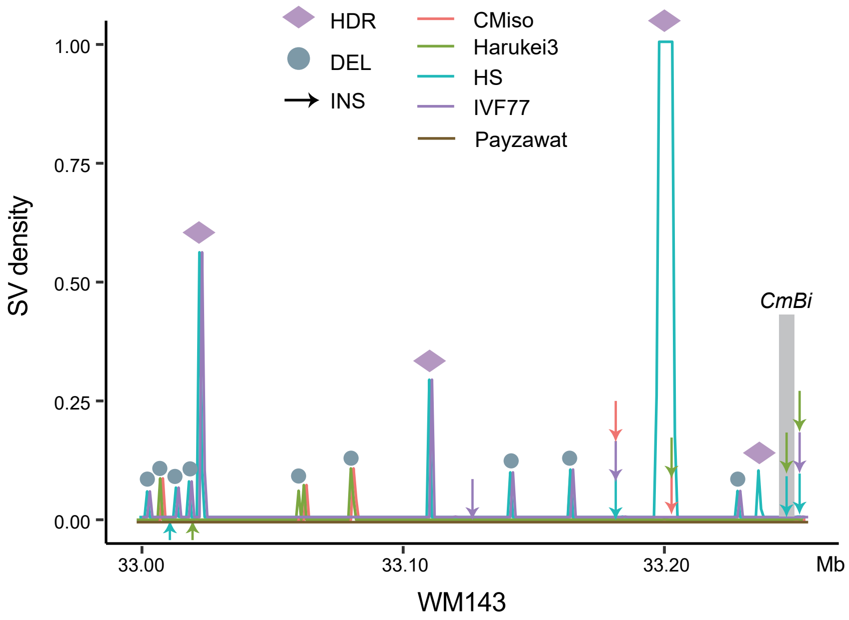

Figure. S10 The SV types and statistics within the WM143 region.

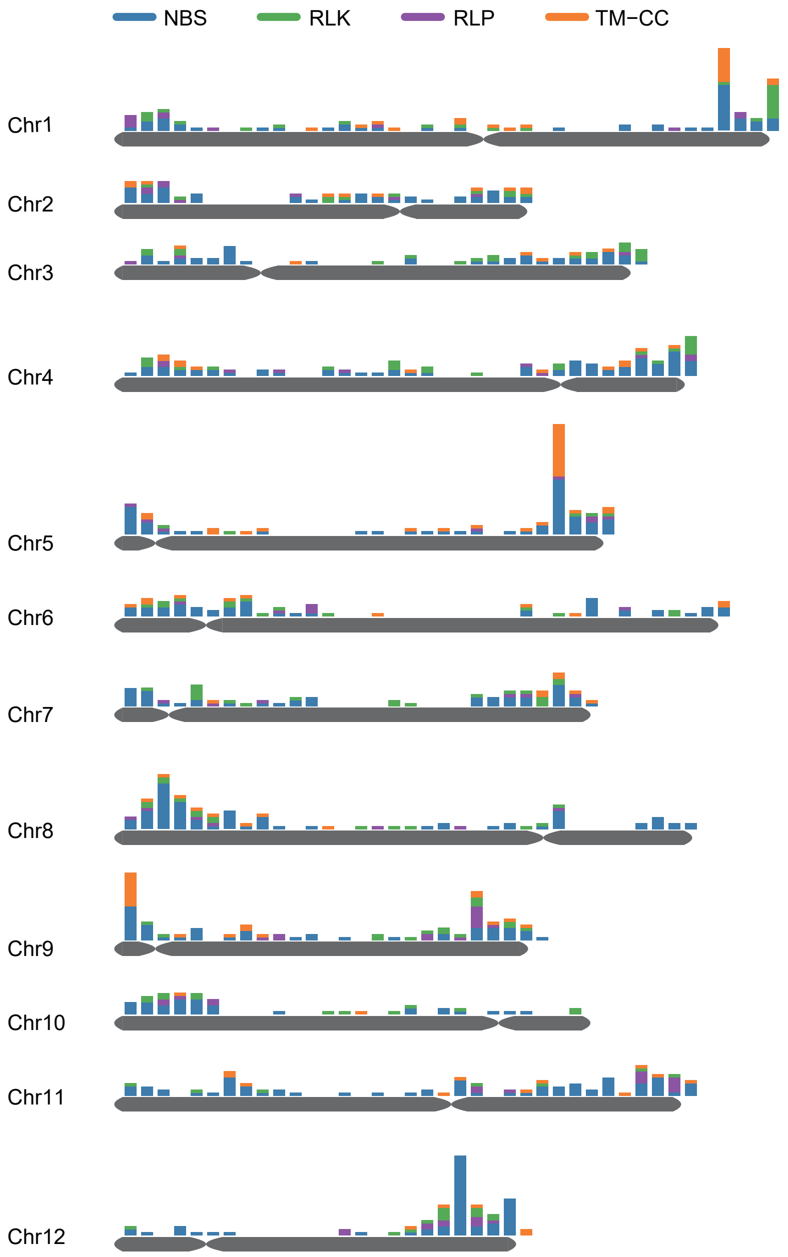

Figure. S11 Distribution of resistance genes on each chromosomes (821_Hap1).

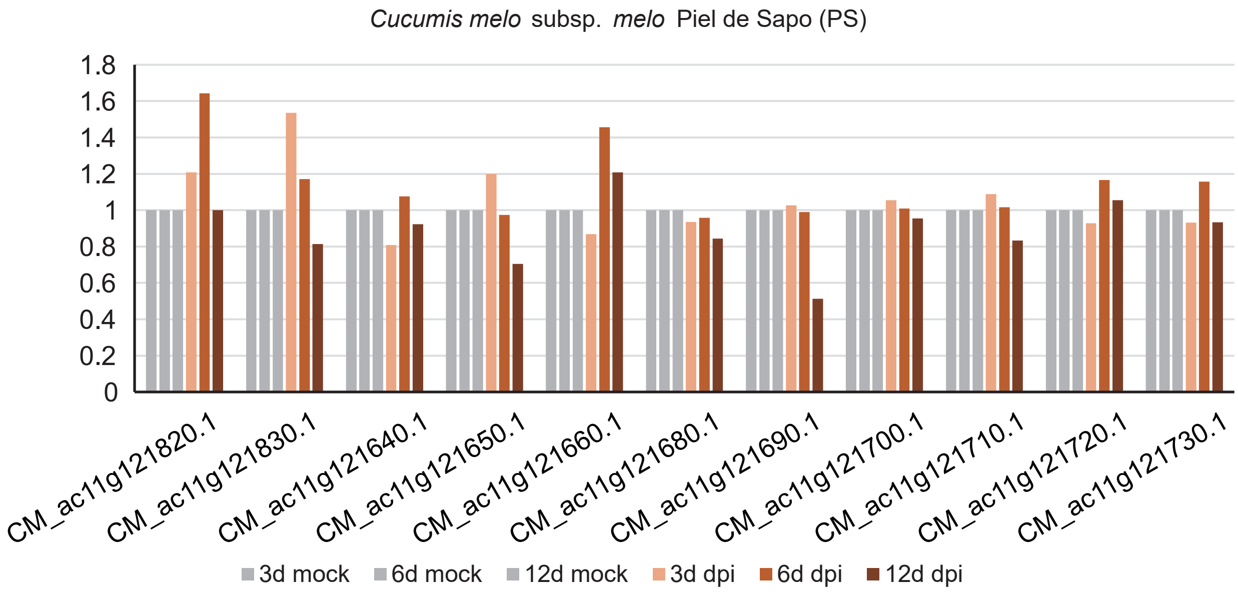

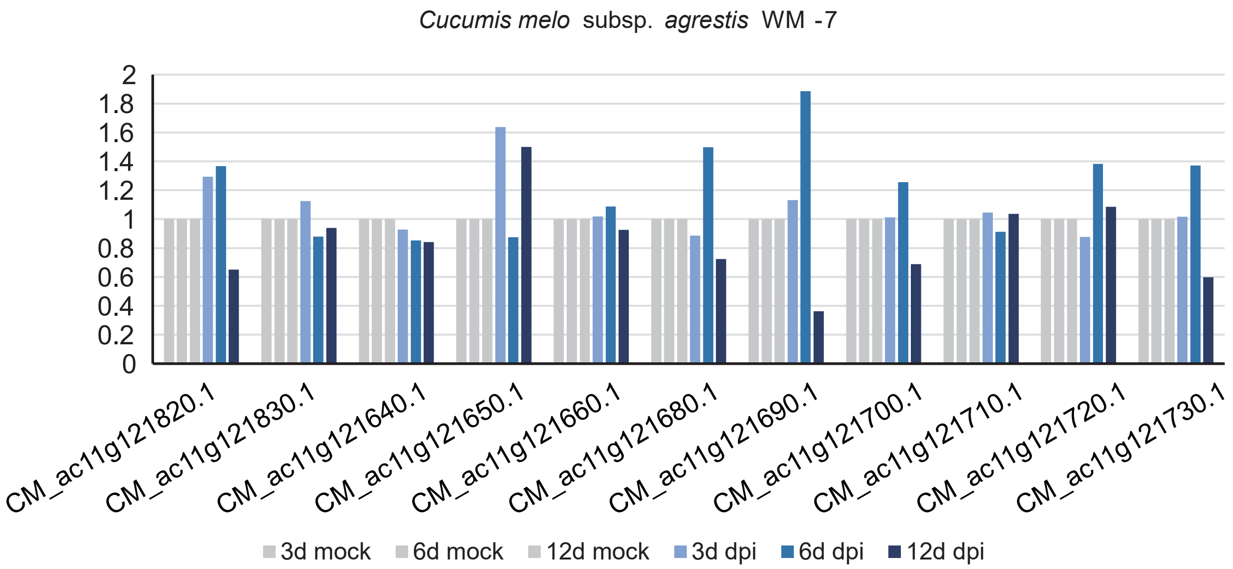

Figure. S12 The different gene expression levels on resistant and susceptible melon accessions.

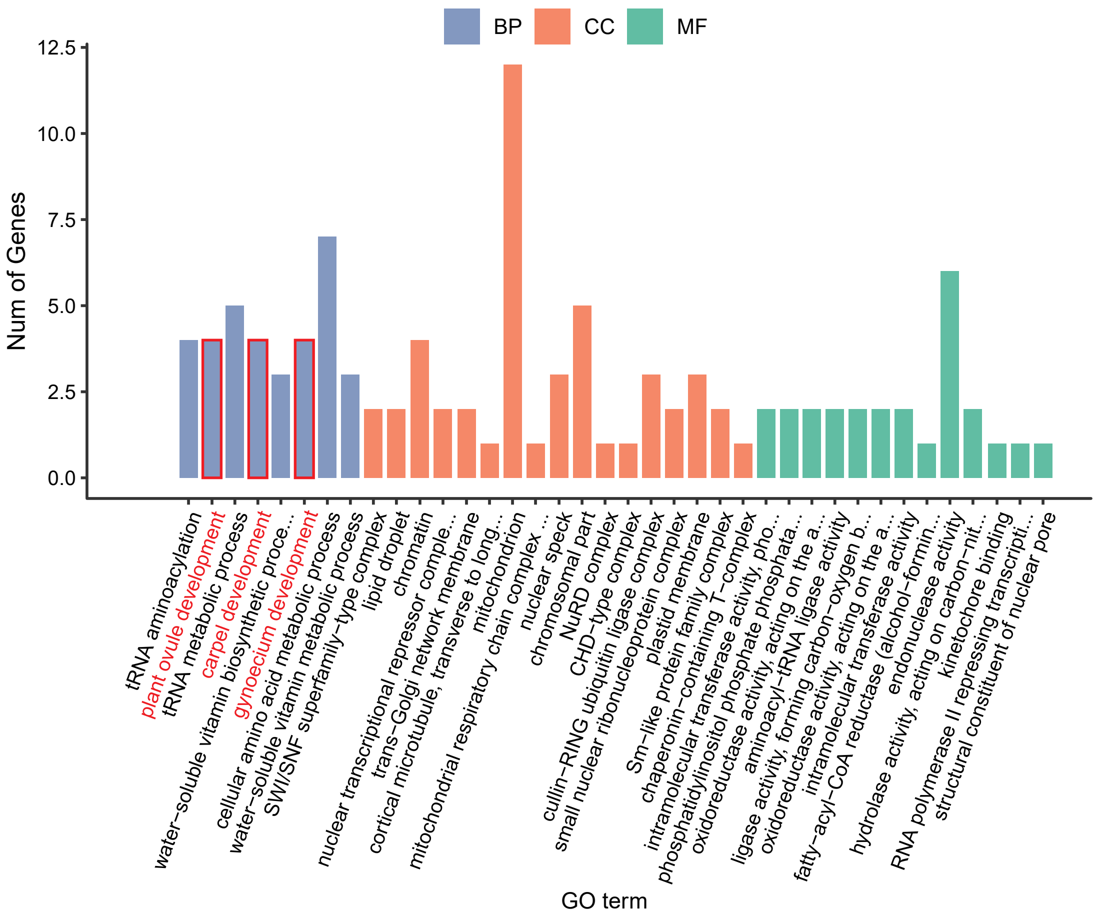

Figure. S13 GO analysis of genes in centromere regions.

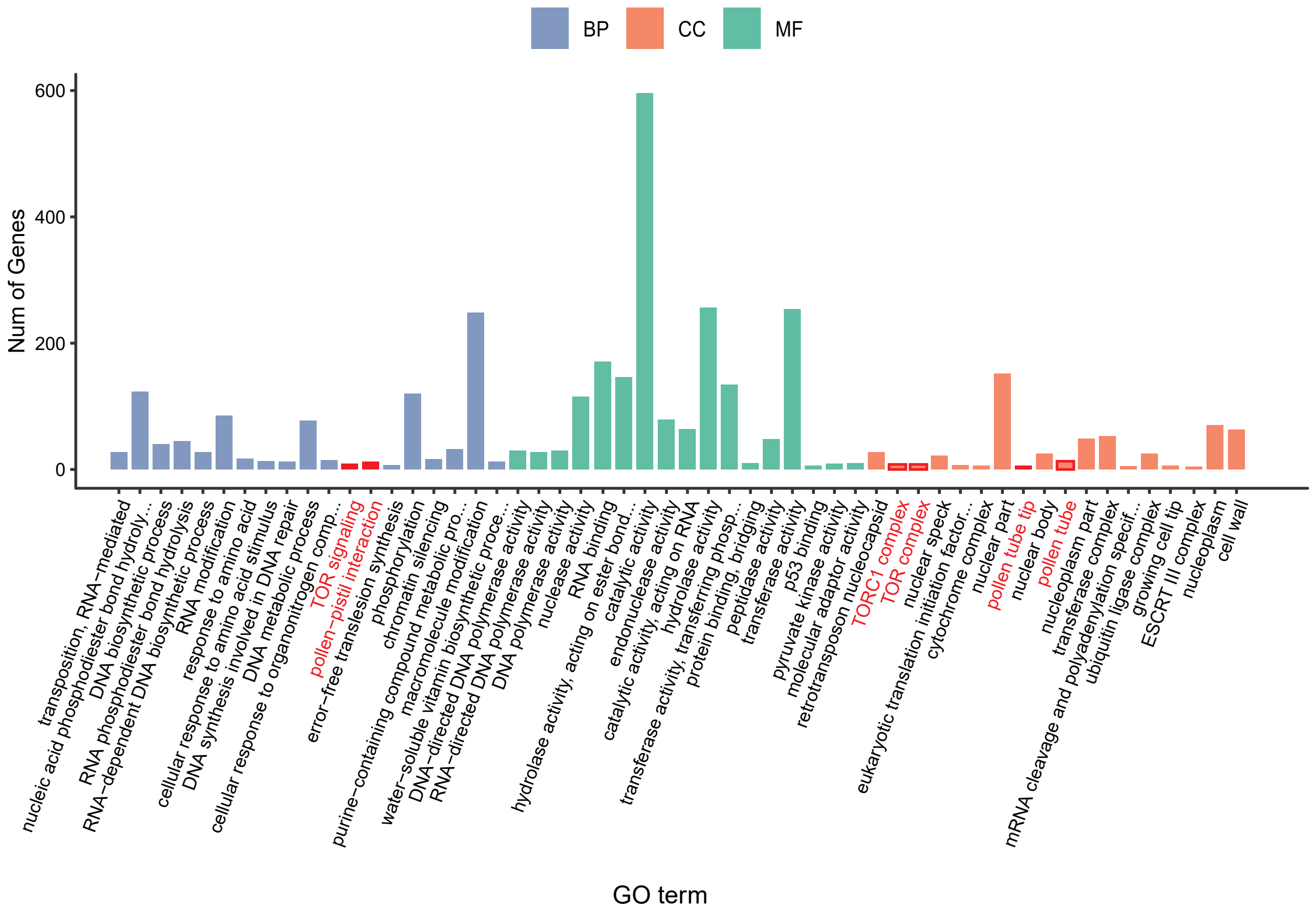

Figure. S14 GO analysis of un-anchor genes of 821_Hap1.
